# Phage cocktail-loaded hydrogel coating prevents the biofilm formation of MDR *Acinetobacter baumannii* on indwelling medical devices

**DOI:** 10.3389/fmicb.2026.1990693

**Published:** 2026-10-30

**Authors:** Nisha K. Rai, Kokkarambath Vannadil Suchithra, Juliet Roshini Mohan Raj, Prakash Patil, B. Mohana Kumar, Veena A. Shetty

**Affiliations:** 1 Department of Microbiology, KS Hegde Medical Academy (KSHEMA), Nitte (Deemed to be University), Deralakatte, Mangalore, India; 2 Department of Infectious Diseases and Microbial Genomics, Nitte University Centre for Science Education and Research, Nitte (Deemed to be University), Deralakatte, Mangalore, India; 3 Central Research Laboratory, KS Hegde Medical Academy (KSHEMA), Nitte (Deemed to be University), Deralakatte, Mangalore, India; 4 Nitte University Centre for Stem Cell Research and Regenerative Medicine, KS Hegde Medical Academy (KSHEMA), Nitte (Deemed to be University), Deralakatte, Mangalore, India

**Keywords:** *Acinetobacter baumannii*, antibiofilm, bacteriophage, hydrogel, medical devices

## Abstract

**Introduction:**

Indwelling catheters and implantable medical devices are vital in clinical care, but are often complicated by device-associated infections, especially caused by *Acinetobacter baumannii*. This pathogen readily colonizes device surfaces and forms biofilms, limiting the efficacy of current preventive strategies such as antimicrobial coatings and surface modifications.

**Methods:**

To address this, we developed a sodium alginate and polyvinyl alcohol-based hydrogel loaded with a bacteriophage cocktail against MDR *Acinetobacter baumannii*. Physicochemical characterisation of the hydrogel was tested, and the gel was coated onto the medical devices, including urinary catheter and Endotracheal tube.

**Results & discussion:**

The system exhibited good biostability of the encapsulated phages and its sustained release behaviour, supporting the biomedical potential. The *in vitro* antibacterial assay demonstrated a clear zone of bacterial inhibition on the tested agar plates, and the antibiofilm assay showed a 4-5 log reduction in bacterial count in the Foley’s urinary catheter and a 4-7 log reduction on endotracheal tube surfaces at their respective physiological pH. In summary, the developed SA-PVA bacteriophage cocktail-loaded hydrogel exhibited strong antibacterial and antibiofilm efficacy along with sustained phage release, highlighting its potential as a translational strategy for preventing MDR *A. baumannii*-associated device-related infections.

## Graphical abstract

**Figure:**
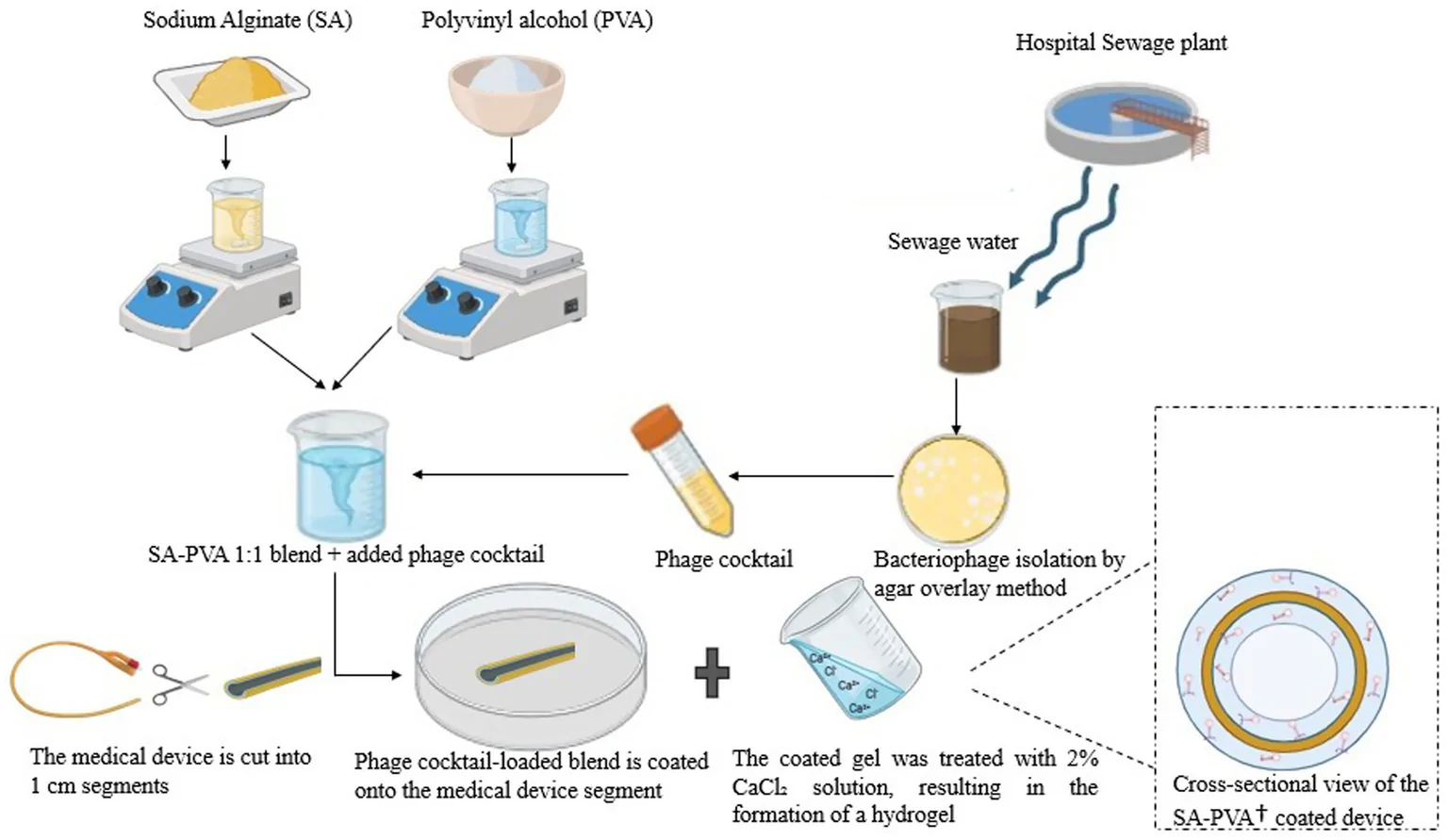

## Introduction

Healthcare-associated infections (HAIs), particularly device-associated infections, continue to be a significant concern for patient safety across the globe, placing a heavy burden on healthcare systems due to increased illness, mortality, and financial costs (CDC - Center for Disease Control and Prevention, 2015). Despite advances in hospital hygiene and infection control protocols, HAIs continue to pose problems in intensive care units, where patients often undergo invasive procedures and depend on life support devices (Blot et al., 2020).

The majority of HAIs are associated with the use of medical devices and are referred to as device-associated healthcare-associated infections. The most commonly reported of these are ventilator-associated pneumonia (VAP), Central line-associated bloodstream infections (CLABSI), and catheter-associated urinary tract infections (CAUTI) (Khan et al., 2022). The use of indwelling devices alters the normal host defence mechanisms and helps microbes to attach to the surface and grow as colonies, thus increasing the risk of infection. Medical devices are responsible for approximately 60–70% of nosocomial infections, raising a major clinical concern (Bouhrour et al., 2024).

Urinary tract infections (UTIs) are one of the most commonly encountered HAIs, responsible for nearly 40% of all cases. Approximately 70—80% of these infections are attributed to indwelling urinary catheters, making catheter-associated Urinary tract infections (CAUTIs) among the most frequent and preventable complications in healthcare environments (Oumer et al., 2021). The duration of the catheterisation is directly proportional to the risk of infection development. Research indicates that almost all patients with an indwelling catheter for more than a month often develop significant bacteriuria with >10^5^ colony-forming units per millilitre of urine (Nicolle, 2014). Proper maintenance of the catheter, combined with its timely removal, significantly lowers infection rates (Parry et al., 2013).

Biofilm formation on the catheter surface plays a crucial role in the development and persistence of infection. Biofilms are complex microbial communities embedded within a self-produced extracellular matrix that holds the bacterial colonies together and attaches them to device surfaces. Mature biofilms act as a protective shield, restricting the entry of antimicrobial agents and helping microorganisms to evade host immune responses (Karygianni et al., 2020). Thus, biofilm-associated infections are challenging to treat and have a high chance of recurrence. They continue to present significant challenges and remain a major priority for hospital infection control teams.

*Acinetobacter baumannii* is a gram-negative coccobacillus often considered a difficult-to-treat pathogen due to its multidrug resistance (MDR), strong biofilm-forming capacity, and ability to persist in hospital environments (Gedefie et al., 2021). This opportunistic bacterium is frequently associated with device-related infections, leading to chronic and persistent infections (Moubareck and Halat, 2020). Despite advances in antimicrobial strategies, conventional device coatings often exhibit poor efficacy against resistant organisms and fail to prevent biofilm formation, highlighting the need for more effective, focused approaches.

Bacteriophages (phages) are considered a promising alternative to antibiotics. They can act against MDR pathogens, as bacterial resistance mechanisms against phages differ from those associated with antibiotics. Their efficacy as antibacterial agents are further highlighted by their specificity and capacity to self-amplify at the site of infection (Uddin Md Uddin et al., 2018). Prior research has shown that they can effectively target and eradicate bacterial populations, including those resistant to traditional antibiotics. Phages also disrupt biofilms and lower the bacterial burden in chronic wounds, thereby indirectly aiding tissue repair (Fanaei Pirlar et al., 2022; Huang et al., 2026). The use of bacteriophages targeting MDR opportunistic bacteria such as *A. baumannii, which is frequently associated with* hospital-acquired and device-related infections, therefore represents a promising therapeutic approach (Jin et al., 2012).

While conventional antibiotic therapies follow standardised dosing and treatment timelines, establishing an optimal delivery protocol for bacteriophages remains a significant challenge. Currently, local delivery via injection or drain tubes provides immediate access to the site of infection (Bessems et al., 2024; Ferry et al., 2022). However, these methods often fail to maintain sustained therapeutic phage concentrations over time (Nilsson, 2019; Onallah et al., 2023).

To overcome these limitations, the current study explored the use of hydrogels as an effective delivery platform owing to their biocompatibility and highly hydrated three-dimensional polymeric network, which enables substantial water retention. Hydrogels represent highly effective delivery systems for the localised and sustained release of bacteriophages due to their high-water content and porous three-dimensional structure (Kim et al., 2021). Among various biomaterials, sodium alginate (SA) and polyvinyl alcohol (PVA) are widely used for hydrogel fabrication because of their excellent biocompatibility and mechanical stability.

A previous study has successfully encapsulated bacteriophages within SA–PVA hydrogel matrices, demonstrating their efficacy as antimicrobial dressings for localised wound infection management (Boggione et al., 2021; Kaur et al., 2019). While these hybrid networks have been predominantly evaluated for topical applications, their ability to facilitate stable immobilisation and prolonged biological activity suggests significant potential for use as bioactive coatings on medical device surfaces to prevent biofilm formation.

Therefore, the present study aimed to develop and characterise an SA–PVA bacteriophage cocktail-loaded hydrogel targeting MDR *A. baumannii* and to evaluate its antibacterial and antibiofilm efficacy for potential application in preventing device-associated infections. Furthermore, the use of a phage cocktail targeting MDR *A. baumannii* through multiple infection pathways enhances antibacterial efficacy and broadens the host range, ensuring more comprehensive targeting and improved bacterial clearance.

## Methods

### Bacterial strains and culture conditions

*Acinetobacter baumannii* ATCC *19606* was used as the host bacteria for phage-related experiments. *Acinetobacter baumannii* ATCC *19606* was routinely grown on nutrient agar (NA, Himedia) or Nutrient broth (NB) at 37 °C, unless specified otherwise.

### Isolation and characterization of bacteriophages targeted to *A. baumannii*

*A. baumannii* bacteriophages vB_AbaM_561a and vB_AbaS_561b, previously isolated from sewage water samples collected from Justice KS Hegde Charitable Hospital (JKSHCH) using standard phage isolation techniques, were characterized for their lytic activity against MDR *A. baumannii*. The purified phages were combined at an equal 1:1 ratio to prepare the phage cocktail for subsequent hydrogel encapsulation studies. The basic characteristics of the individual phages are provided in the Supplementary Information SI1.

### Preparation of phage cocktail-loaded SA-PVA hydrogel (SA-PVA†^1^)

A sodium alginate (SA)- polyvinyl alcohol (PVA) composite hydrogel was prepared via ionic crosslinking using CaCl_2_ (SA-PVA), augmented by physical crosslinking from freeze–thaw procedures. Briefly, a 2% (w/v) SA solution was made by melting the polymer powder in sterile distilled water under mild heat and constant stirring to yield a uniform, transparent blend. Similarly, an 8% (w/v) PVA solution was obtained by fully dissolving PVA in distilled water through gentle heating and persistent agitation. These individual solutions were autoclaved, then cooled to room temperature before subsequent steps. After sterilization, both the semisolid solutions were thoroughly mixed in different ratios (1:2, 1:1, 2:1) under aseptic conditions to guarantee homogeneity. In addition to the composite formulations, polymer membranes containing pure SA and pure PVA were prepared separately using the respective polymers under the same processing conditions.

For the preparation of SA-PVA†, bacteriophage cocktail (10^9^ PFU/mL) was blended into the polymer mixture with light stirring for even diffusion of the phages, preserving their activity. The phage concentration used in the formulation was selected based on the previously performed checkerboard assay evaluating the combined activity of the two bacteriophages against MDR *A. baumannii*. Considering an anticipated approximately 1-log reduction in phage titre during the hydrogel formulation process and storage, a higher initial concentration of 10^9^ PFU/mL was used for phage loading. The device segments were dip-coated in the gel mixture. In case of Foley’s urinary catheter made of latex, the device was immersed for a period of 15 min for uniform coating, and the endotracheal tube was dipped for three cycles, each for 15 min. As the ET tube is made of PVC material, it requires more coating cycles. Then, it was subjected to 3–4 freeze–thaw cycles (12 h at −20 °C, followed by 4 h thawing at room temperature) to aid in PVA crystallization and physical crosslinking (Kaur et al., 2019; Li et al., 2018). 2% (w/v) CaCl_2_ was poured gently onto the coated devices to support the ionic crosslinking of the alginate, followed by overnight incubation to allow complete crosslinking. The residual CaCl_2_ was removed by washing in sterile distilled water. The final resulting solid thin film of the hydrogel-coated device was kept at 4 °C for further characterization.

### Morphological and physicochemical characterisation of hydrogels

#### SEM analysis

Scanning electron microscopy (SEM) was used to gain insights into the surface morphology of the prepared hydrogels. For this, freeze-dried samples, including crosslinked hydrogel, hydrogels without crosslinker and the phage-encapsulated hydrogels, were affixed to the aluminium stubs with conductive adhesive and given a thin gold sputter-coating to minimise charging effects. The structural morphology, porosity, and structural stability of the hydrogels were assessed using a Zeiss EVO 10 Scanning Electron Microscope.

#### FTIR analysis

Fourier transform infrared (FTIR) spectroscopy was employed to identify functional groups and bonding interactions within the SA-PVA hydrogel, SA-PVA-CaCl_2_ hydrogel, and their individual pure form of hydrogels. The analysis was carried out at the wavenumber range of 400-4000 cm^−1^ using a Bruker Alpha II FTIR spectrometer.

#### Mechanical property

The mechanical properties of the hydrogels were evaluated using a Universal Testing Machine. Measurements were carried out for hydrogels before and after CaCl₂-mediated crosslinking, along with the phage-loaded hydrogel. The test hydrogels were cut into uniform dumbbell-shaped specimens (approximately 15 mm width, 30 mm length, and 0.06 mm thickness), and each hydrogel piece was mounted between the grips of the instrument and subjected to tensile loading at a constant crosshead speed of 100 mm/min until failure. The tensile strength, along with strain at break, was measured from the obtained records of applied force and corresponding displacement, and the tensile behaviour was characterised in a stress–strain curve.

#### Swelling behaviour

The swelling behaviour of the hydrogel was evaluated by a gravimetric method. Briefly, the hydrogel film was cut into 1 × 1 cm pieces and dried at room temperature until a constant weight was obtained. The initial dry weight of each sample (W_0_) was recorded using an analytical balance. The dried hydrogel samples were then immersed in PBS at both acidic (pH 6) and neutral pH (pH 7.5) conditions and incubated at 37 °C under static conditions. After 24 h, the samples were removed from the PBS, and excess surface water was gently blotted using tissue paper. The swollen weight of each sample (W_s_) was then measured (Khan et al., 2023).

The swelling index was calculated using the following equation:

$$\frac{\mathrm{Ws}-\mathrm{Wo}}{\mathrm{Wo}}X1\mathrm{OO}$$

Where *Wo* represents the initial dry weight, and *Ws* denotes the weight of the swollen hydrogel at the respective time point.

#### Degradation study

The degradation behaviour of the SA-PVA and SA-PVA† was assessed in phosphate-buffered saline (PBS) at different pH conditions, including acidic and neutral pH. Briefly, the freeze-dried samples (1 × 1 cm) were weighed (W_o)_ and incubated in PBS (pH 7.5) at 37 °C with gentle shaking. At days 1, 3, 5, and 7, samples were removed, rinsed with distilled water, freeze-dried, and reweighed (W_t_) to determine the remaining mass. A similar degradation study was carried out in PBS adjusted to an acidic pH of 6, mimicking urinary tract pH conditions (Li et al., 2026).

The degradation profile was expressed as remaining weight percentage using the following equation.

Remaining weight (%) = (W_t_/W_o_) × 100.

All experiments were performed in triplicate.

### Stability assay

The stability of the bacteriophage-loaded hydrogel was evaluated during storage at 4 °C and 25 °C (Lab temperature) for a period of 5 weeks. Samples were analyzed at weekly intervals (1–5 weeks). At each time point, hydrogels were incubated under shaking conditions in SM buffer for 1 h to elute phages, and the recovered lysate was serially diluted and quantified by agar overlay plaque assay against *A. baumannii.*

#### Bacteriophage release assay

Bacteriophage release behaviour from the hydrogel was studied under acidic pH 6 and neutral pH 7.5 environments through a diffusion-based assay. Briefly, a 1 × 1 cm piece of hydrogel was immersed in 1 mL of PBS and maintained at 37 °C under gentle agitation. At selected intervals (1, 2, 4, 6, 12, 24, 48, 72, and 96 h), a 100 μL aliquot of the release medium was withdrawn and immediately replaced with 100 μL of fresh PBS to maintain a constant release volume of 1 mL.

The collected samples were serially diluted, and the number of released bacteriophages was determined using double-layer agar plaque assay with the appropriate host bacteria. The phage concentration was expressed as PFU/mL, and the amount of active phage released per unit surface area was calculated and expressed as PFU/cm^2^. The total amount of phage released over experimental time was determined and plotted to assess the release kinetics. All experiments were performed in triplicate.

### Antibacterial and antibiofilm properties of hydrogels

#### Antibacterial assay

To evaluate the antibacterial efficacy of the hydrogel formulations, we employed two methods: an agar-based diffusion assay and a growth inhibition assay.

In agar diffusion assay, confluent bacterial lawns were prepared by using overnight bacterial cultures adjusted to a 0.5 McFarland standard and uniformly spread onto Mueller-Hinton agar plates. Sterile 1 × 1 cm pieces of phage cocktail-loaded hydrogel were carefully placed onto the inoculated agar surface. Hydrogel without phage served as a negative control, while the phage cocktail lysate was directly spotted onto the bacterial lawn as a positive control. The plates were incubated at 37 °C for 18–24 h. The antibacterial activity was assessed by observing zones of inhibition or clear lytic areas surrounding the hydrogel samples.

In the growth inhibition test, hydrogel alone and phage cocktail-loaded hydrogel pieces were added to the tube containing BHI broth inoculated with an overnight bacterial culture. For comparison, in another tube, bacterial culture was treated with phage cocktail lysate directly without any hydrogel. The control tube contained only the bacteria without any treatment. Following an 18–24 h incubation at 37 °C, the tubes were visually inspected for turbidity. A clear tube indicates successful bacterial inhibition, while a cloudy (turbid) tube indicates bacterial growth (Jumaa et al., 2002).

### Antibiofilm testing and bacterial colony count

Catheter and endotracheal tube segments were employed to assess the antibiofilm activity of the SA-PVA hydrogel. The medical devices were cut into 1 cm sections, coated with the hydrogel formulation, and freeze-dried prior to use. An overnight culture of *A. baumannii* grown in nutrient broth was introduced to each well of a sterile 12-well plate containing the coated device segments. The following groups were included in the experimental setup: (i) untreated device pieces (negative control), (ii) device pieces treated with phage cocktail lysate (10^9^ PFU/mL) as a positive control, (iii) device pieces coated with hydrogel without phage, and (iv) device pieces coated with bacteriophage cocktail-loaded hydrogel (treatment group).

Two experimental environments were employed to mimic physiological circumstances. In order to simulate urinary conditions, the medium pH for Foley catheter assays was set to 6, and this pH was maintained in related control and treatment groups. For endotracheal tube testing, pH 7.5 was set to represent the near-neutral physiological conditions.

Following incubation at 37 °C for 24 h, the medium was removed, and the device pieces were gently washed with sterile PBS to eliminate planktonic bacteria. The pieces were then transferred to tubes containing 1 mL sterile saline and sonicated (20 kHz, 10s, three cycles) to detach biofilm-associated cells. The resultant suspensions were serially diluted, plated on nutrient agar, and incubated at 37 °C for 24 h. Colonies were counted, and results were expressed as log_10_ CFU per device piece.

To further validate biofilm formation and its inhibition, surface morphology was examined using Scanning Electron Microscopy. Hydrogel-coated device segments, hydrogel without phage (control), and phage-loaded hydrogel-treated samples were exposed to bacterial culture and incubated for both 24 and 48 h. Following incubation, the samples were processed and imaged to visualize biofilm architecture and confirm the antibiofilm efficacy of the phage-loaded hydrogel.

### Human cell biocompatibility/indirect cytotoxicity evaluation

#### Indirect cytotoxicity assay (MTT assay)

The cytocompatibility of the bacteriophage cocktail-loaded hydrogel was evaluated in a human Gingival fibroblast cell line using the MTT assay. Fibroblast cells were maintained in Dulbecco’s Modified Eagle’s Medium (DMEM) supplemented with 10% fetal bovine serum and incubated at 37 °C in a humidified atmosphere containing 5% CO_2_.

Cells were seeded in 96-well plates at a density of 10,000 cells/well and allowed to attach for 24 h. After incubation, the culture medium was replaced with fresh medium containing the phage cocktail, hydrogel and phage cocktail-loaded hydrogel extract, while untreated cells served as the control. The cells were incubated for 24 and 48 h. Following treatment, the wells were gently rinsed with PBS, and 100 μL of MTT solution (1 mg/mL) was added to each well. The plates were incubated for 3–4 h at 37 °C to allow the formation of formazan crystals. The MTT solution was then removed, and the crystals were dissolved using DMSO. Absorbance was measured at 570 nm using a microplate reader. Cell viability was calculated relative to the control group. All experiments were performed in triplicate.

#### Colony formation assay (Clonogenic assay)

To assess the colony-forming ability of the fibroblast cells, the cells were seeded at a density of 5 × 10^2^ cells in a 6-well culture plate and allowed to adhere overnight with DMEM media, incubated in a 5% CO_2_ incubator. The following day, the cells were exposed to treatment conditions: untreated control, hydrogel matrix, phage cocktail-loaded hydrogel and the phage cocktail alone. The cells were maintained for 14 days with periodic media and treatment replacement to permit colony development. At the end of the incubation period, the conditioned media were decanted, and the cells were fixed with 3.4% paraformaldehyde and stained with 0.5% crystal violet. The colonies comprising at least 50 cells were counted to determine colony-forming ability and compared with the control group.

#### Dual staining (AO/EB) for the detection of apoptotic cells

Fibroblast cell apoptosis was further confirmed using Acridine orange/Ethidium Bromide (AO/EtBr) dual staining with slight modifications to established methods (Kasibhatla et al., 2006). Cells were seeded at a density of 10^5^ cells per well in a 12-well plate and allowed to attach under standard culture conditions (37 °C, 5%CO_2_). The treatment group cells were treated with hydrogel alone or bacteriophage-loaded hydrogel, and the cells without any treatment served as a control group. After incubating for 24 h and 48 h, the medium was removed, and the cells were gently washed with PBS. Cells were fixed using methanol for 30 min at room temperature, followed by PBS washing. AO/EtBr solution (10 mg/mL each) was added directly to the attached cells and incubated for 10 min in the dark. Excess stain was removed with PBS. Cells were immediately observed under the Zoe fluorescent cell imager, and at least 200 cells per sample were analyzed.

#### Assessment of apoptosis by flow cytometry

Fibroblast cells were treated with phage cocktail, hydrogel alone, and phage cocktail-loaded hydrogel for 24 and 48 h. Following treatment, cells were harvested using trypsin, washed with PBS, and the cell pellet was resuspended in Annexin V binding buffer. eBioscience™ Annexin V-FITC apoptosis detection kit (Invitrogen, CA 92008 United States) was used according to the manufacturer’s instructions for the analysis. Cells were first stained with Annexin V-FITC (5 μL) and then with Propidium iodide (5 μL). After incubation for 20 min at room temperature in the dark, cells were subjected to flow cytometry (CytoFLEX, Beckman Coulter) and apoptosis was analysed using CytExpert software.

### Statistical analysis

GraphPad Prism 8.0.2 was used for all statistical analysis of the data generated in the study. All experiments were conducted in triplicate, and the results are expressed as mean ± standard deviation (SD). The antibiofilm and cytocompatibility assays were analysed using one-way ANOVA with Dunnett’s multiple comparison test and two-way ANOVA for the grouped analysis. Differences were considered statistically significant at *p* < 0.05.

## Result

### SEM analysis exhibiting the microporous architecture of the hydrogel

In Figure 1. The crosslinked SA-PVA hydrogel displayed a distinct three-dimensional architecture composed of interconnected pores of mean size 8 μm with a moderately rough surface texture. This morphology is indicative of effective ionic crosslinking between Ca2 + ions and the carboxyl groups of alginates, leading to the formation of a stable and organized network. Similarly, the bacteriophage cocktail-loaded hydrogel also maintained the porous network with a mean pore size of 8.5 μm, suggesting that phage incorporation did not compromise the gel structure. However, minor morphological variations were evident, including slightly enhanced surface irregularities and localised pore occupancy, which may reflect the presence and distribution of bacteriophages within the matrix.

**Figure 1:**
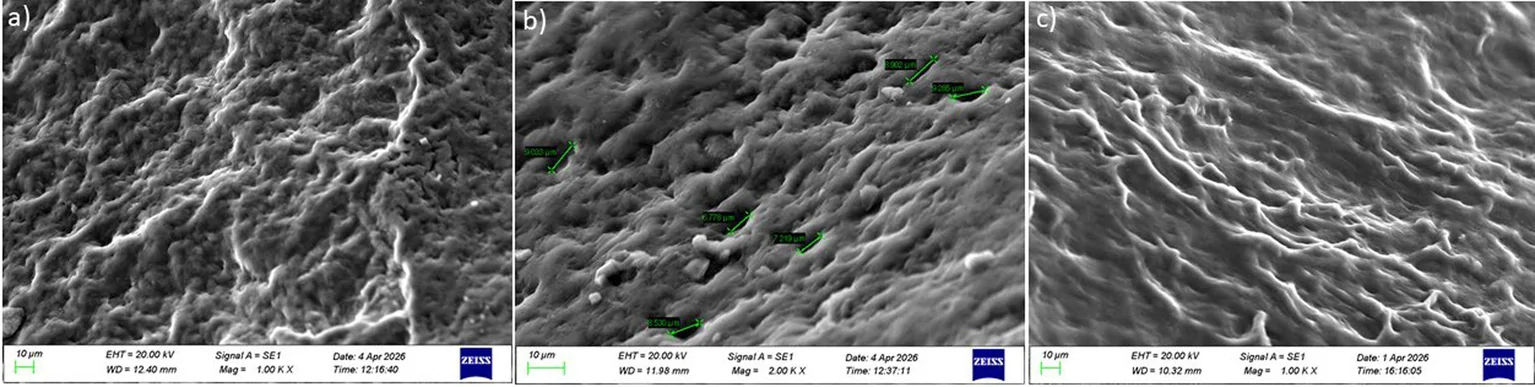
SEM micrographs of SA-PVA-based hydrogel displaying its structural morphology. **(a)** Crosslinked SA-PVA hydrogel exhibiting a compact interconnected network, **(b,c)** bacteriophage-loaded cross-linked hydrogel displaying phage-incorporated porous morphology. Scale bar: 10 μm.

### FTIR confirms the stable SA-PVA hydrogel network formation

The FTIR spectra confirmed the characteristic features and interactions of hydrogel components (Figure 2A). Sodium alginate alone displayed polysaccharide peaks with prominent bands at 1597 cm^−1^ and 1,419 cm^−1^, corresponding to -COO^−^ groups, indicating the presence of alginate in its sodium salt form rather than the carboxylic acid form. The peak shift suggests chemical modification or cross-linking. In the polyvinyl alcohol (PVA) alone hydrogel, the spectrum showed features characteristic of a hydroxyl-rich, hydrophilic polymer, enabling hydrogen bonding with alginate in blends. A pronounced peak at 1637 cm^−1^ indicates bound water interfering with carbonyl bonds. In the SA + PVA blend, the O-H band broadened and shifted to 3335.17 cm^−1^ compared to SA and PVA alone, indicating hydrogen bonding between alginate and PVA. The absence of new peaks suggests physical blending instead of chemical modification. It is observed that after the addition of CaCl_2_ to the SA-PVA blend, the shift and intensity change of the carboxylate band from 1597.93 to 1637.08 cm^−1^ confirms the ionic coordination with alginate -COO^−^ groups, indicating Na + substitution by Ca^2+^ and supporting formation of the characteristic egg-box crosslinked network structure. The retention of characteristic PVA peaks confirms its physical incorporation within the calcium-crosslinked alginate network.

### Crosslinking improves mechanical integrity of SA–PVA hydrogels

The stress–strain curve presented in Figure 2B demonstrates the mechanical behaviour of the hydrogel formulations. The SA PVA hydrogel without crosslinker mixing exhibited relatively low mechanical strength, with a maximum stress of 1.22 MPa and elongation at break of 14.4%. Upon crosslinking, a marked improvement in mechanical performance was observed, with the tensile strength increasing to 3.58 MPa and the strain at break rising to 22.1%, indicating enhanced network integrity and flexibility. In contrast, the phage-loaded crosslinked hydrogel showed a negligible reduction in tensile strength (3.51 MPa) along with a notable decrease in elongation (10.56%), suggesting that phage incorporation affects the deformability of the network.

**Figure 2:**
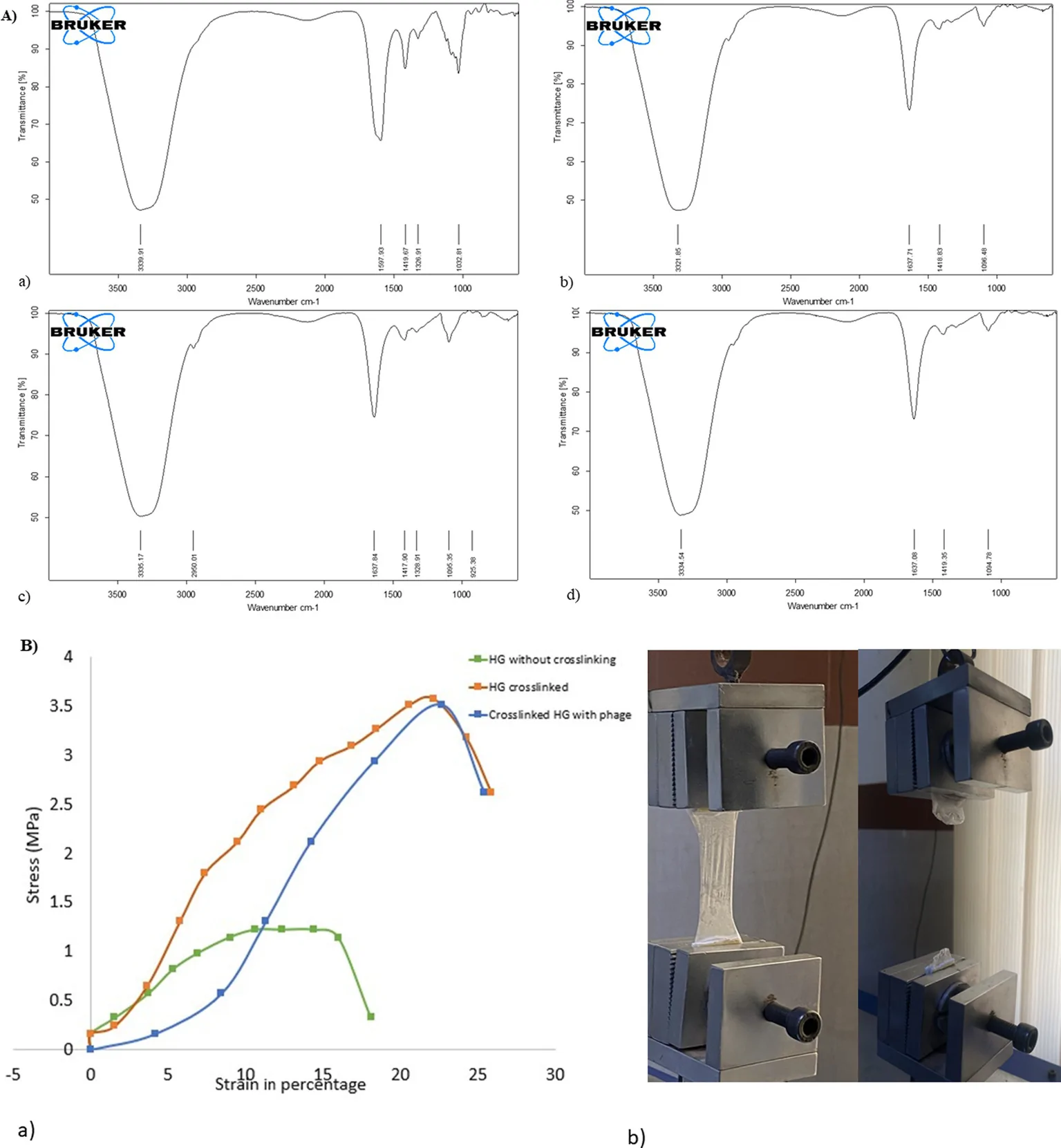
**(A)** Fourier-transform infrared (FTIR) spectra of (a) Sodium alginate (SA), (b) Polyvinyl alcohol (PVA), (c) SA-PVA hydrogel, and (d) SA-PVA hydrogel crosslinked with calcium chloride (CaCl_2_). The spectra reveal the distinct functional groups of the individual polymers along with their interactions in the composite hydrogel system. **(B)** Mechanical properties of the hydrogel. a) The stress–strain behaviour of hydrogel samples under tensile loading. The crosslinked hydrogel exhibits higher stiffness and maximum stress than the uncrosslinked sample, indicating improved strength due to network formation. The phage gel follows a similar trend but exhibits slightly reduced mechanical performance. (b) In the Universal testing machine, the hydrogel was fixed between the grips and subjected to uniaxial tensile loading until failure.

Consistent with these observations, the calculated Young’s modulus was highest for the crosslinked hydrogel (17 MPa), followed by the non-crosslinked hydrogel (15 MPa), while the phage-loaded hydrogel exhibited the lowest modulus (13.6 MPa), indicating a reduction in stiffness upon phage incorporation. Overall, the stress–strain profiles further corroborated these findings, with the crosslinked hydrogel displaying a steeper slope and higher resistance to deformation compared to other formulations.

### Alginate-rich hydrogels exhibit greater swelling capacity

The swelling capacity of the hydrogels differed under neutral and acidic pH conditions, depending on the polymer composition (Figure 3A). At neutral pH, pure sodium alginate showed the highest swelling index of 813.2%, whereas the pure PVA hydrogel exhibited a comparatively lower swelling index, recording 513.9%. Among the blended formulations, the 2:1 SA: PVA ratio exhibited the greatest swelling, followed by the 1:1 blend with moderate swelling of 716.5% and the 1:2 blend with comparatively lower swelling. These observations indicate that the incorporation of sodium alginate contributes significantly to the water uptake capacity of the hydrogel matrix. Under acidic conditions, all the formulations showed reduced swelling compared to neutral pH. Based on the experiment, the 1:1 SA: PVA formulation was chosen for further characterization, as its moderate swelling behaviour is suitable for coating medical device surfaces.

**Figure 3:**
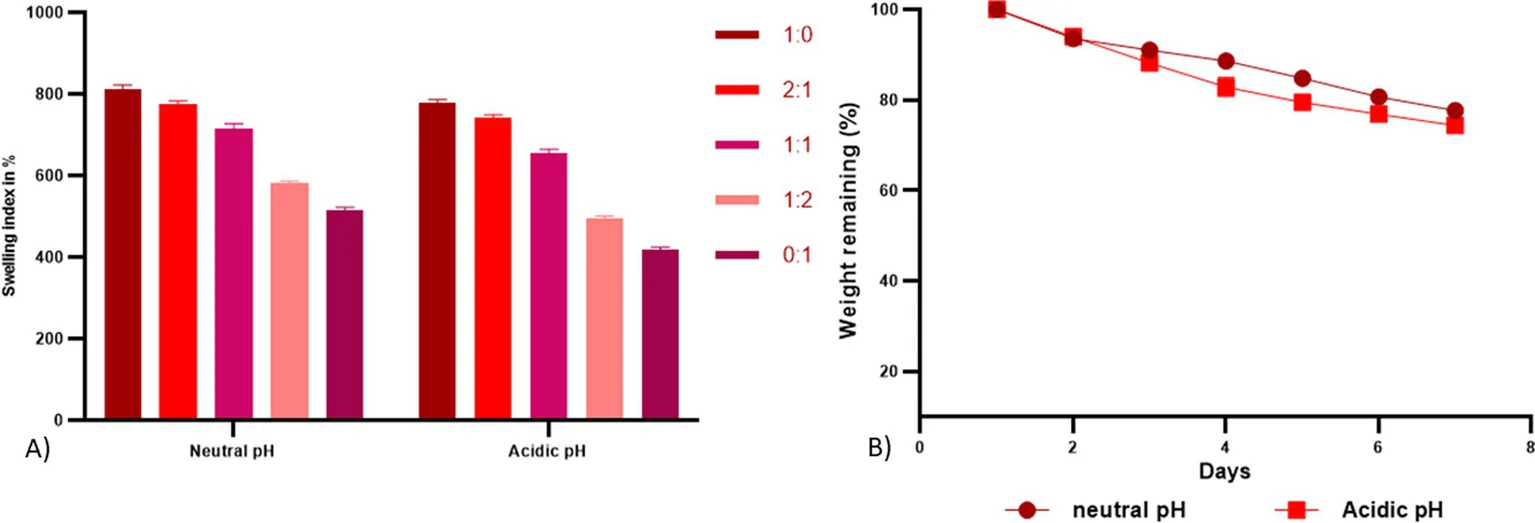
**(A)** Swelling behaviour of the hydrogel with different blend ratios was assessed under acidic and neutral pH conditions and expressed as swelling index percentage. **(B)** Degradation profile of the SA PVA hydrogel assessed under acidic and neutral pH conditions over a period of 7 days. The data is represented as the percentage of remaining hydrogel weight.

### Controlled degradation of SA–PVA hydrogels in physiological and acidic environment

The hydrogel demonstrated a gradual increase in degradation under both pH conditions during the 7-day duration (Figure 3B). Under acidic conditions, the degradation occurred at a noticeably higher rate, with a marked increase in mass loss evident by day 3, followed by further acceleration until the end of the study. However, hydrogels maintained at neutral pH exhibited a slower, more gradual degradation profile, with comparatively lower weight loss throughout the incubation period. A continuous decline in dry mass from day 1 through day 7 reflected the ongoing hydrolytic disintegration of the polymer matrix.

### Hydrogel-entrapped bacteriophages remain stable during Long-term storage

The stability of bacteriophages incorporated within the hydrogel was assessed over a period of 5 weeks using a plaque assay. The phage-loaded Hydrogel was stored at 4 °C and 25 °C, and phage titres were measured at weekly intervals to assess the stability under both conditions (Figure 4A).

**Figure 4:**
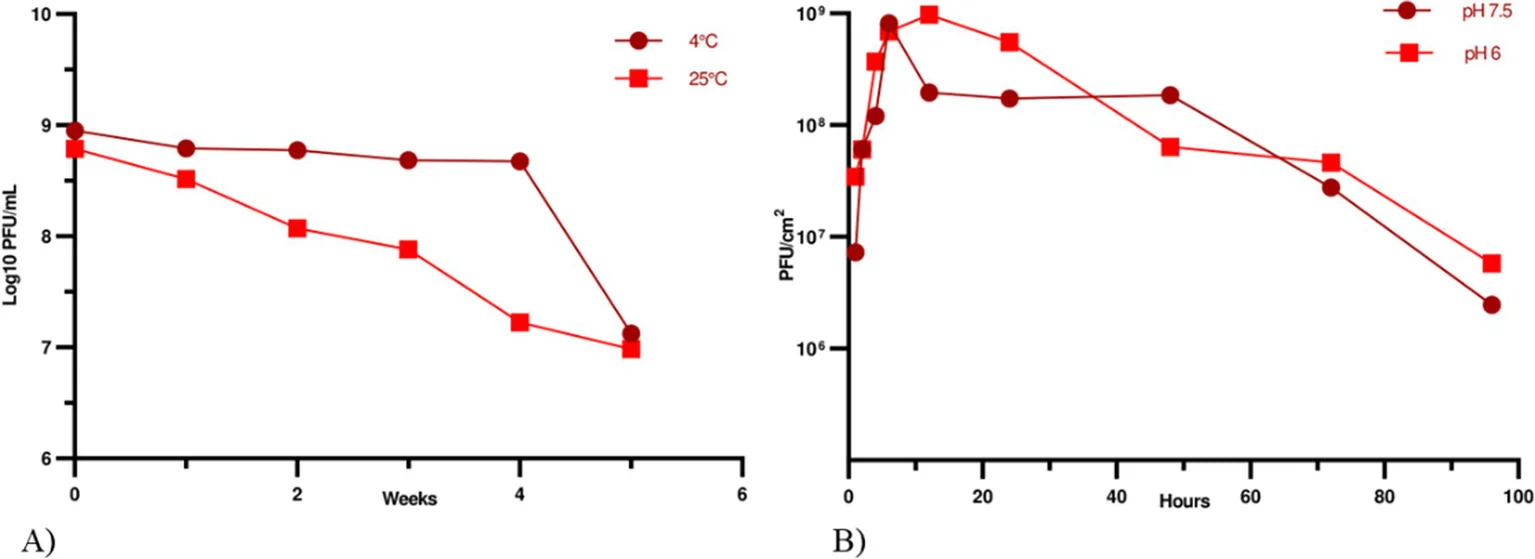
**(A)** Stability analysis of bacteriophages encapsulated within the SA-PVA hydrogel during storage at 4 °C and 25 °C over a period of 5 weeks. Phage viability was evaluated at weekly intervals using plaque assay, and the titres are represented as log10 PFU/mL. **(B)** *In vitro* bacteriophage release profile from the phage cocktail-loaded hydrogel coating on the 1 cm-long medical device surface. Phage release was evaluated at 37 °C under two pH conditions: pH 7.5 and pH 6. The total phage release per unit surface area at each time point was measured to assess the effect of environmental pH on release kinetics, demonstrating a pH-dependent release pattern.

Under refrigerated conditions (4 °C), the phage titre showed a steady but moderate decline over time. The initial mean phage titer recorded on day 1 was 8.95 log10 PFU/mL, which gradually decreased over the next 5 weeks and in the 5th week, the titre was 7.12 log10 PFU/mL. Although a gradual decline in phage titer was observed during storage, the total reduction remained less than one log unit.

However, samples stored at 25 °C demonstrated a more pronounced reduction in phage viability, with titres decreasing from 8.78 log10 PFU/mL on day 1 to 6.98 log10 PFU/mL by the fifth week, indicating that higher storage temperatures may adversely affect phage stability.

### pH-responsive sustained release of bacteriophages from the hydrogel matrix

Figure 4B shows the *in vitro* bacteriophage release from the crosslinked hydrogel composite. It was evaluated at 37 °C under neutral and acidic pH conditions to stimulate physiological and infection-relevant environments. At neutral pH, the hydrogel exhibited a gradual release profile, with approximately 79% phage release observed at 6 h.

In contrast, under acidic conditions, a comparatively higher release rate was observed, with nearly 85% phage release at 6 h. Furthermore, the highest phage titres were observed between 6 and 24 h, ranging from 7 to 9.7 × 10^8^ PFU/cm^2^. These findings indicate a clear pH-dependent release behaviour, with a more efficient and greater release observed at acidic pH compared to neutral conditions. The enhanced phage release observed under acidic conditions may be associated with increased protonation of ionisable functional groups within the polymeric matrix, resulting in partial disintegration of the gel network and subsequent release of the entrapped active agents.

In contrast, under acidic conditions, a comparatively higher release rate was observed, with nearly 85% phage release at 6 h. Furthermore, the highest phage titres were observed between 6 and 24 h, ranging from 7 to 9.7 × 10^8^ PFU/cm^2^. These findings indicate a clear pH-dependent release behaviour, with greater phage release observed under acidic conditions compared with neutral conditions. The enhanced phage release observed under acidic conditions may be associated with increased protonation of ionisable functional groups within the polymeric matrix, resulting in partial disintegration of the gel network and subsequent release of the entrapped active agents.

### Phage-loaded hydrogel exhibits potent antibacterial activity against *A. baumannii*

The antibacterial performance of the prepared hydrogels against *A. baumannii* was investigated using the agar diffusion assay and the antibacterial tube method. In the agar diffusion test, observations made after 24 h of incubation showed that the hydrogel without any loaded phages did not produce any inhibition zone, indicating that the hydrogel itself lacks antibacterial properties. In comparison, the direct addition of bacteriophage lysate generated a distinct, clear zone, demonstrating effective lytic activity against the bacterial strain. Notably, the phage-loaded hydrogel produced the largest inhibition zone after 24 h, indicating the successful and sustained release of active bacteriophages from the hydrogel matrix (Figure 5A).

**Figure 5:**
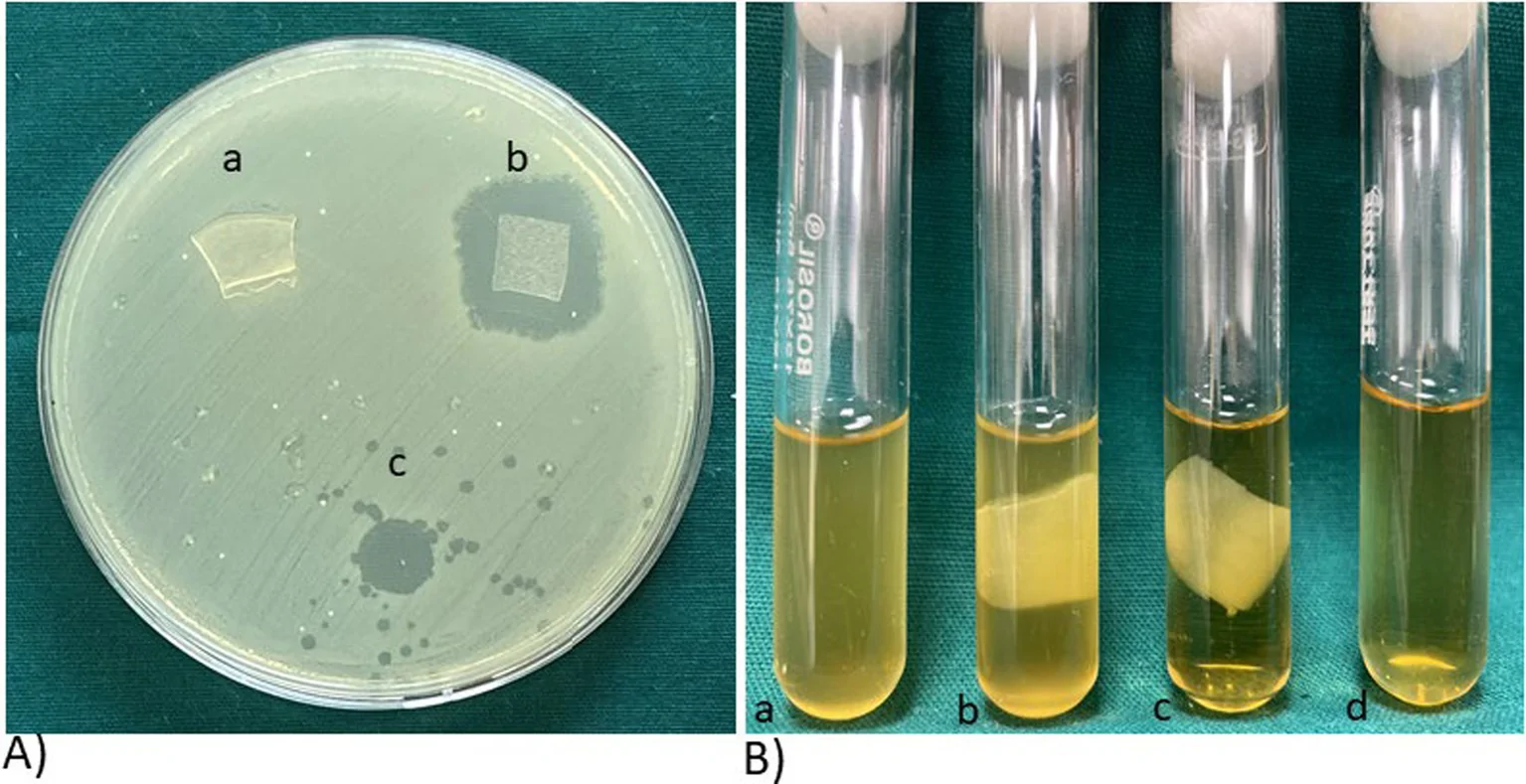
Antibacterial activity of bacteriophage-loaded hydrogel against ATCC *A. baumannii* strain. **(A)** Agar plate assay: 1 × 1 cm of a) hydrogel (alone), **(B)** phage cocktail-loaded hydrogel and c) phage cocktail lysate was directly placed on an *A. baumannii* lawn and incubated for 18–24 h. No inhibition zone was seen with hydrogel alone, while clear zones appeared with phage lysate and phage-loaded hydrogel. **(B)**. Growth inhibition assay: In BHI broth, (a) the control tube containing only the bacteria and (b) hydrogel alone showed turbidity (growth), whereas (c) phage-loaded hydrogel and (d) phage lysate resulted in clear cultures (no growth), with stronger activity observed for the phage-loaded hydrogel.

Similar results were obtained from the growth inhibition assay. After 24 h incubation, the tube containing unloaded hydrogel became turbid, reflecting active bacterial growth. In contrast, the tubes treated with bacteriophage lysate and phage-incorporated hydrogel remained clear, suggesting effective inhibition of bacterial proliferation. These findings demonstrate that the phage-loaded hydrogel exhibits significant antibacterial activity against *A. baumannii* (Figure 5B).

### Phage-loaded hydrogel effectively Controls *A. baumannii* biofilms on the tested medical device surfaces

The antibiofilm efficacy of the bacteriophage cocktail-loaded hydrogel was evaluated on two clinically relevant medical device materials: latex urinary catheters and the endotracheal tube made of Polyvinyl chloride (Figure 6A). The assessment was performed following incubation periods of 24 h and 48 h under physiologically relevant pH conditions, with Foley catheter assays maintained at pH 6 and endotracheal tube assays at pH 7.5. Biofilm formation was compared across four treatment conditions: (i) untreated control, (ii) hydrogel-coated surfaces, (iii) phage lysate-treated surfaces, and (iv) phage-loaded hydrogel-coated surfaces (Figure 6B).

**Figure 6:**
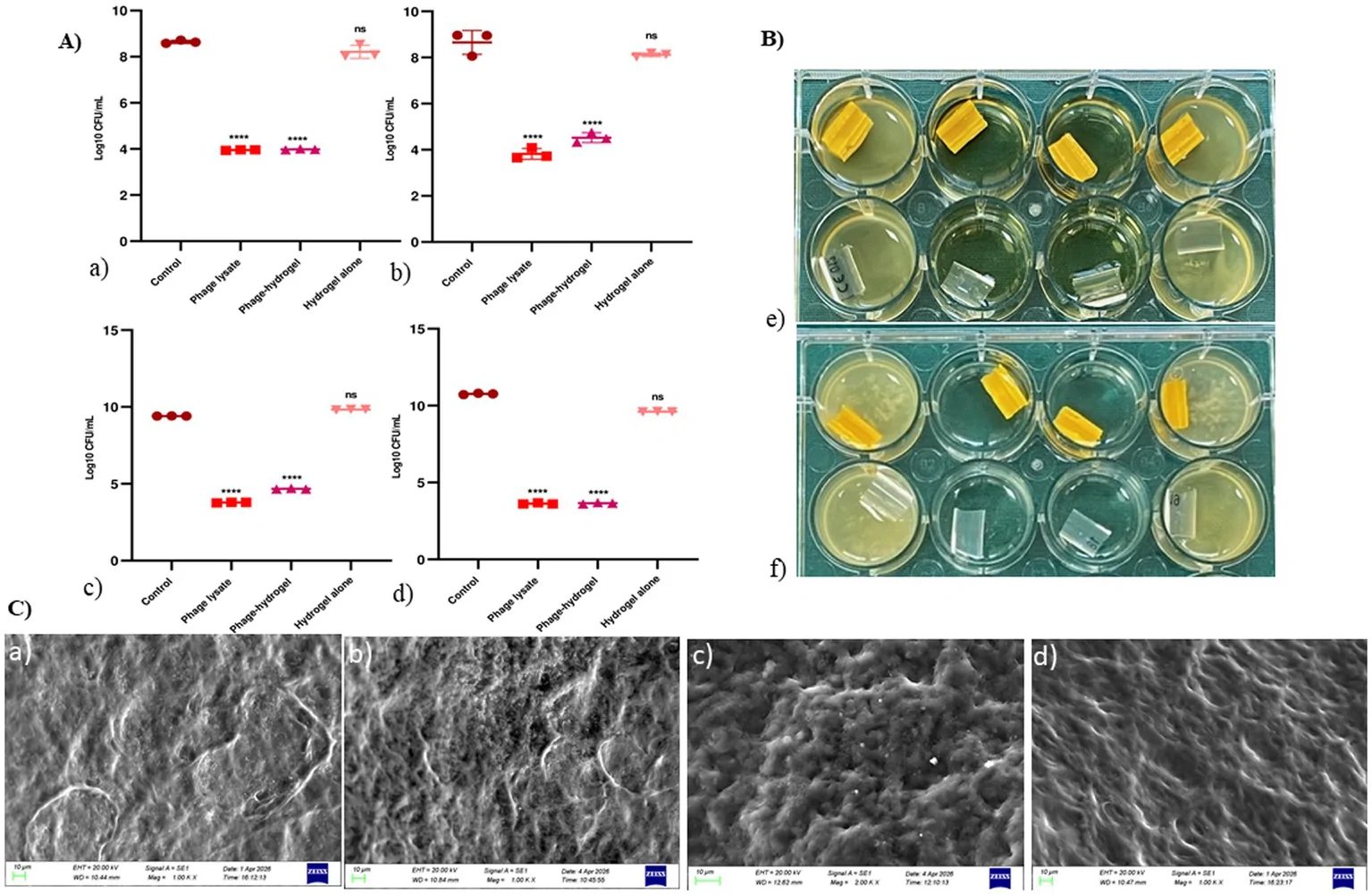
Antibiofilm activity of the SA-PVA† hydrogel-coated medical device segments against *A. baumannii* evaluated by viable colony count analysis. Untreated control and phage cocktail lysate-treated controls were used for the relative comparison. **(A)** a, b indicates the antibiofilm activity of the coated urinary catheter and endotracheal tube segments treated for 24 h, respectively, with a significant reduction in CFU count. **(A)** c, d show the significant antibiofilm activity of the respective devices with treatment for 48 h. **(B)** e, f shows the representative images of the 12-well plates used for the 24 and 48 h assays, respectively, showing differences in turbidity among the treatment groups compared to the control wells. Data are presented as mean ± SD. **(C)** SEM images showing antibiofilm activity of the phage cocktail-loaded hydrogel after 48 h of treatment. (a) Foley urinary catheter and (b) Endotracheal tube control surfaces without any treatment showing dense biofilm formation and extensive bacterial colonization. (c) Foley urinary catheter and (d) Endotracheal tube surfaces treated with phage-loaded hydrogel showing disrupted biofilm architecture, reduced bacterial adhesion, and minimal residual biofilm. Scale bar-10 μm.

At 24 h, the untreated urinary catheter segments exhibited a bacterial load of 8.64 log10 CFU/mL. In contrast, the catheter segments treated with phage-loaded hydrogel demonstrated a significant reduction of approximately 4 log units in bacterial count (*p* < 0.0001), which was comparable to the reduction observed with direct bacteriophage lysate treatment. A similar trend was observed in endotracheal tubes, where phage-loaded hydrogel coating resulted in a 4-log reduction in bacterial burden compared to the untreated control (*p* < 0.0001).

At 48 h, further reductions in bacterial load were observed. The phage-loaded hydrogel-coated catheter segments showed a ~ 5 log reduction (*p* < 0.0001), while the coated endotracheal tubes exhibited a ~ 7 log reduction (*p* < 0.0001) in CFU/mL relative to the untreated control. Notably, surfaces coated with hydrogel alone showed bacterial counts comparable to the untreated control in all cases, indicating that the hydrogel matrix itself does not possess intrinsic antibacterial activity.

Scanning electron microscopy (SEM) analysis further validated the antibiofilm efficacy of the bacteriophage cocktail-loaded hydrogel-coated devices against *Acinetobacter baumannii*. Consistent with the CFU findings, Figure 6C shows that the control device coated with hydrogel alone exhibited dense, well-organised biofilm architecture with extensive bacterial colonization on the surface. In contrast, devices coated with the phage-loaded hydrogel showed a marked disruption of biofilm structure at 24 h, characterized by reduced bacterial adhesion and compromised cell morphology. This effect was more pronounced at 48 h, with substantial depletion of bacterial cells and minimal residual biofilm, indicating progressive and sustained antibiofilm activity of the phage-loaded system.

### Cytocompatibility assessment of phage-loaded hydrogels in human gingival fibroblast cells

#### Phage-hydrogel formulation maintains fibroblast viability

The cytocompatibility of the phage cocktail-loaded hydrogel was assessed using an MTT assay in Human Gingival Fibroblast cells after 24 and 48 h of incubation (Figure 7A). The treated groups exhibited cell viability levels comparable to the untreated control at both time points, remaining near baseline values. A minor decrease in viability was noted at 48 h; however, this change was not statistically significant (*p* > 0.05). These results suggest that the hydrogel formulations are non-cytotoxic and well-tolerated by the human fibroblast cells.

**Figure 7:**
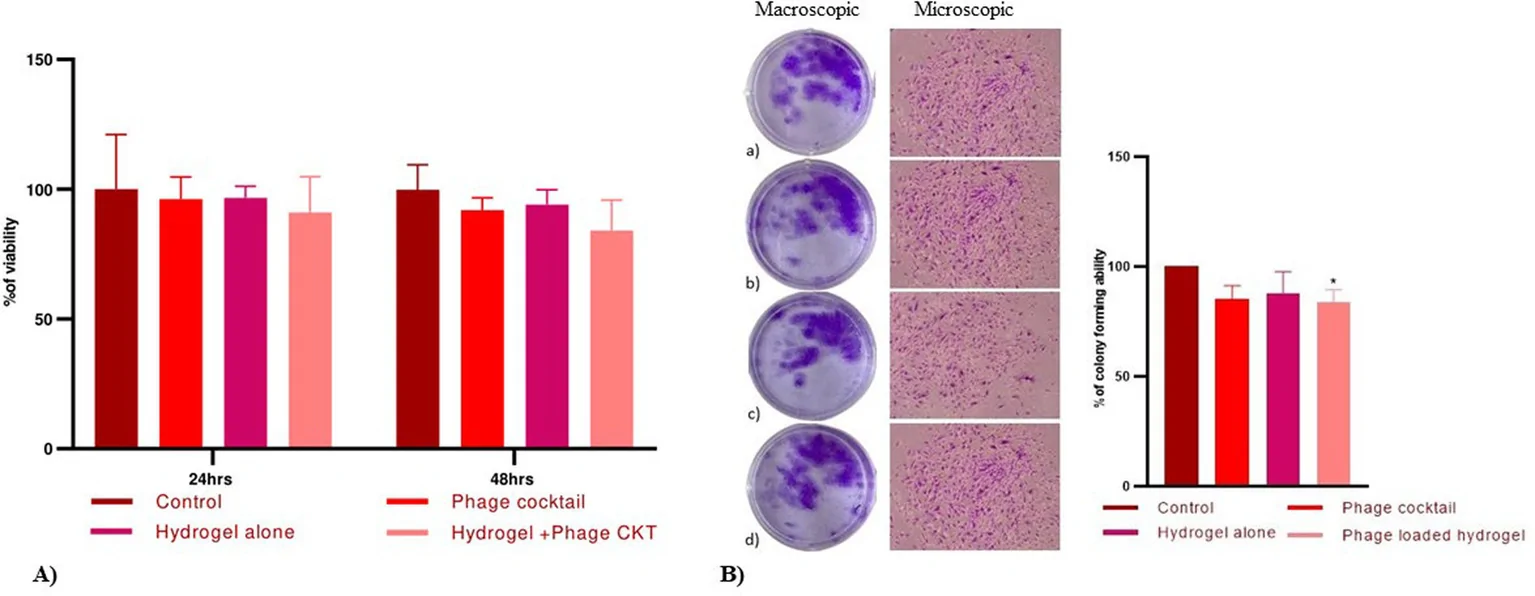
**(A)** MTT assay of human fibroblast cells treated with phage cocktail, hydrogel, and phage-loaded hydrogel extracts for 24 and 48 h. Cell viability remained comparable to the control, indicating good cytocompatibility. **(B)** Clonogenic potential of human fibroblast cells treated with phage cocktail and hydrogel. Left: representative macroscopic and microscopic images of colony formation after 14 days with crystal violet staining: (a) control, (b) phage cocktail, (c) hydrogel, and (d) phage-loaded hydrogel. Right: quantification of colony formation relative to the control. Data are presented as mean ± SD from three independent experiments. Statistical analysis was performed by one-way ANOVA, where * indicates *p* < 0.05 vs. control.

#### Colony-forming ability of the fibroblast cells are well maintained by the phage hydrogel

The assay demonstrated that the human fibroblast cells were capable of forming colonies on 10–14 days following treatment with the phage cocktail, hydrogel alone, and phage-loaded hydrogel (Figure 7B). Representative macroscopic and microscopic images revealed comparable homogeneous population of clonal cell morphology and cellular distribution across all groups. Quantitative analysis showed no significant reduction in colony formation in the bacteriophage cocktail (*p*-value = 0.05) and hydrogel-treated groups (*p*-value = 0.11), with colony formation percentages of 92.3 and 94.8, respectively. However, cells exposed to the phage-loaded hydrogel exhibited a slight but statistically significant decrease (*p*-value = 0.034) in the number of colonies (*p* < 0.05), with a CFU percentage of 87 in relation to the control group. Despite this reduction, substantial colony formation was still observed, indicating that the formulations maintained acceptable cytocompatibility toward human fibroblast cells.

#### Low apoptotic activity in treated cells was confirmed by dual staining and flow cytometry

Acridine orange (AO) and Ethidium Bromide (EB) dual staining was carried out to examine the effect of different treatments on the viability of human fibroblast cells for 24 h (Figure 8A) and 48 h (Figure 8B). The control cells showed predominantly green fluorescence, reflecting intact membranes and normal viability. A similar staining pattern was observed in cells treated with the bacteriophage cocktail and hydrogel alone, with only occasional orange/red signals, indicating minimal apoptotic or necrotic changes. The combined hydrogel-bacteriophage treatment also preserved cell integrity, as most cells remained green with very few showing early apoptotic characteristics. Taken together, these findings suggest that both the individual treatments and their combination are cytocompatible and do not induce significant apoptosis under the experimental conditions.

**Figure 8:**
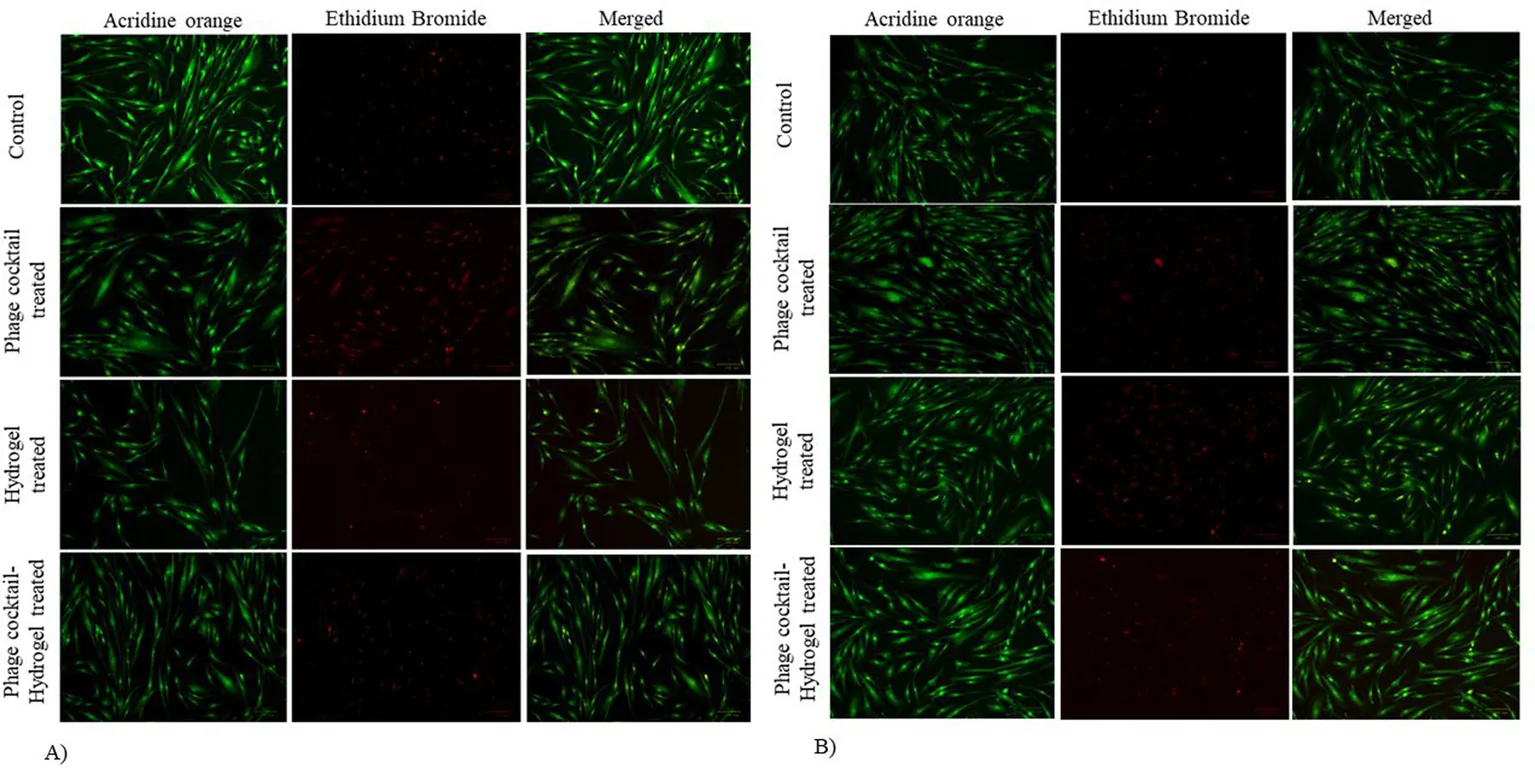
AO/EB live-dead staining analysis of apoptosis in human gingival fibroblast cells after 24 & 48 h of treatment. **(A)** Cells after 24 h of treatment. **(B)** Cells after 48 h of treatment. Predominant green fluorescence represents viable cells, whereas orange/red fluorescence indicates apoptotic cells. The high proportion of viable cells with minimal orange/red fluorescence indicates good cytocompatibility of the phage cocktail, hydrogel and phage-loaded hydrogel. Scale bar = 100 μm.

In the Annexin V FITC staining (Figure 9), the untreated control exhibited the highest percentage of viable cells (97.2% at 24 h), with only a small fraction of cells 0.60% & 0.83% apoptotic at 24 h and 48 h. Following treatment with the phage cocktail, hydrogel, and phage-loaded hydrogel for 48 h, the apoptotic cell rate remained at 0.46%, 2.27%, and 1.6%, respectively. The corresponding proportions of apoptotic cells were comparable to those observed in the untreated control, and no statistically significant differences were detected in the treatment group compared to the control group (*p* > 0.05).

**Figure 9:**
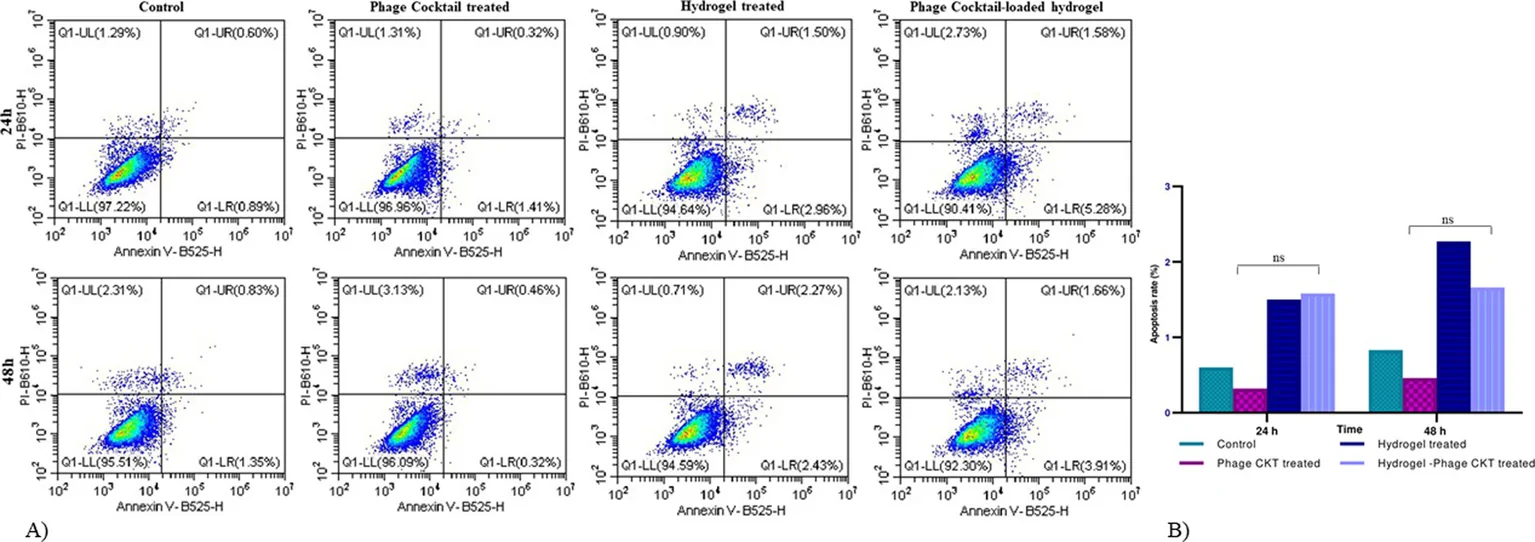
Annexin V-FITC flow cytometric analysis of apoptosis in human gingival fibroblast cells following 24 and 48 h of treatment with the phage cocktail, hydrogel, and phage-loaded hydrogel. **(A)** The lower-left quadrant represents viable cells (Annexin V-/PI-), the lower-right quadrant represents early apoptotic cells (Annexin+/PI-), THE UPPER-right quadrant represents late apoptotic cells (Annexin V+/PI+), and the upper-left quadrant represents necrotic cells (Annexin V-/PI+). **(B)** Corresponding bar graphs show the percentage of apoptotic cells in each treatment group at 24 and 48 h. No significant differences in apoptosis were observed between the treated and untreated groups (*p* > 0.05).

## Discussion

*A. baumannii-*associated medical device infections are difficult to treat, especially when caused by multidrug-resistant (MDR) strains. The organism readily forms persistent biofilms, survives in hospital environments, and colonises indwelling medical devices, thereby reducing antibiotic efficacy and contributing to chronic infections. Due to its remarkable genetic adaptability*, A. baumannii* can acquire multiple resistance mechanisms simultaneously, leading to therapeutic failure (Ayswarya et al., 2026). It is responsible for nearly 22.3% of hospital-acquired pneumonia cases, with carbapenem resistance rates exceeding 70%. The emergence of New Delhi Metallo-β-lactamase (NDM)-producing strains has further complicated treatment options, as these isolates exhibit resistance even to last-resort β-lactam antibiotics (Naseef and Ganesh Pitchaipillai Sankar, 2025). Consequently, alternative therapeutic approaches, such as bacteriophage therapy, are increasingly being explored around the globe.

In the present study, a bacteriophage cocktail-loaded sodium alginate–polyvinyl alcohol (SA-PVA†) hydrogel was developed as a coating for medical device surfaces and evaluated for its antibacterial and antibiofilm activity against MDR *A. baumannii*. The developed system combined the stability and biocompatibility of polymeric hydrogels with the specificity and antibacterial activity of bacteriophages. Importantly, localised delivery of phages directly at the site of infection may reduce the need for repeated administration associated with conventional therapies. In addition, the use of a phage cocktail broadens host range and may minimise the emergence of phage-resistant bacterial populations.

Hydrogels containing antimicrobial agents have been widely investigated for biomedical applications, including wound healing, tissue engineering, and localised drug delivery (Langer and Tirrell, 2004). Previous studies have demonstrated the successful incorporation of bacteriophages into hydrogel systems for managing biofilm-associated infections. Walji et al. (2025) reported a poly (oligoethylene glycol methacrylate)-based hydrogel capable of localised phage delivery against *Pseudomonas aeruginosa* burn wound infections. Similarly, phage-functionalized catheter coatings *reduced Staphylococcus epidermidis* biofilm formation, while injectable phage-loaded hydrogels demonstrated antibacterial efficacy in bone infection models (Curtin and Donlan, 2006; Wroe et al., 2020). Other antimicrobial hydrogel systems, including silver nanoparticle-embedded and antimicrobial peptide-loaded formulations, have also shown promising activity against MDR pathogens (Dai et al., 2024; Khan et al., 2019).

The SA-PVA hydrogel system used in this study was selected because of its favourable physicochemical and biological properties. Sodium alginate provides excellent biocompatibility, water absorption capacity, and controlled release characteristics, while PVA contributes mechanical strength, elasticity, and film-forming ability (Abourehab et al., 2022; Chen et al., 2022). Crosslinking using freeze–thaw cycles followed by calcium chloride treatment produced a stable polymeric network while maintaining mild conditions suitable for preserving bacteriophage viability. The resulting hydrogel provided a hydrated and protective environment capable of supporting sustained phage release.

Stability of encapsulated bacteriophages is a critical factor influencing the therapeutic effectiveness of phage delivery systems (Kim et al., 2021). In this study, phage viability remained relatively stable during storage at both 4 °C and 25 °C over 5 weeks, with only a gradual reduction in titre. Approximately one log reduction was observed at 4 °C and two log reductions at 25 °C, indicating that the hydrogel effectively preserved phage activity during storage. Release studies further demonstrated controlled and sustained phage release under both acidic and neutral pH conditions. The released phage concentrations remained sufficient to inhibit bacterial growth, while phage replication in the presence of host bacteria may further enhance antibacterial efficacy and prolonged biofilm control. Thus, the observed results indicate that the SA-PVA hydrogel matrix provides an effective platform for bacteriophage encapsulation and facilitates its application as a coating on medical devices. Incorporation of the active phages within the polymeric film enabled their retention at the coated surface, offering an advantage over the liquid nature of free phage lysate, which may be more readily dispersed from the target site. The hydrogel coating therefore facilitates the site-specific delivery of active phages at appropriate concentrations while limiting their rapid loss into the surrounding environment, promoting prolonged persistence at the device surface.

Hydrogel swelling and degradation play important roles in regulating bacteriophage release from polymeric matrices (Rizwan et al., 2017). In the present study, the selected 1:1 SA-PVA formulation exhibited moderate swelling properties, enabling uniform coating of medical device surfaces without lumen obstruction. Gradual hydrogel degradation supported sustained phage release over time. Greater degradation was observed under acidic conditions compared to neutral pH, likely due to protonation of polymer functional groups and accelerated hydrolytic cleavage of polymer chains, resulting in destabilisation of the polymeric network (Imanga et al., 2023).

Mechanical characterization demonstrated that crosslinking significantly improved tensile strength and structural stability of the SA-PVA hydrogel (Cui et al., 2025). Following bacteriophage incorporation, a slight reduction in tensile strength, elongation, and stiffness was observed, possibly due to disruption of polymer network uniformity by embedded phage particles (Rivera et al., 2025). However, the phage-loaded hydrogel retained sufficient structural integrity for biomedical and device-coating applications. SEM analysis further revealed a porous and interconnected architecture favourable for fluid transport and sustained release, while subtle surface irregularities suggested successful phage incorporation within the polymeric network (Abed et al., 2024).

Biocompatibility remains an essential consideration for antimicrobial hydrogel systems, particularly following incorporation of biologically active agents such as bacteriophages (Barros et al., 2020). In the present study, the phage-loaded hydrogel did not significantly affect fibroblast viability or colony-forming ability compared to controls. AO/EB staining also demonstrated minimal apoptotic or necrotic cell populations, indicating negligible cytotoxicity. These findings suggest that incorporation of bacteriophages within the hydrogel matrix does not compromise cellular compatibility and supports its suitability for biomedical applications (Li et al., 2022).

Previous reports have demonstrated the therapeutic potential of phage-loaded hydrogels in wound healing and biofilm-associated infections (Beschastnov et al., 2023; Yan et al., 2021). Hydrogels provide localised and sustained phage delivery, thereby improving antibacterial efficacy and stability at the site of infection. Chen B. et al. demonstrated that an alginate hydrogel containing bacteriophages and meropenem effectively controlled *P. aeruginosa* fracture-related infections while reducing biofilm formation (Chen et al., 2023). Despite these advances, the application of phage-loaded hydrogels against *A. baumannii* biofilms on medical device surfaces remains poorly explored.

In the present study, the phage-loaded hydrogel coating demonstrated significant antibiofilm activity on urinary catheter and endotracheal tube surfaces under physiologically relevant pH conditions. These findings indicate that the developed coating effectively maintained phage activity across different device environments and may serve as a promising strategy for preventing MDR *A. baumannii* biofilm formation on indwelling medical devices.

Overall, the developed bacteriophage cocktail-loaded SA-PVA hydrogel coating showed favourable functional and biological performance, with effective antibiofilm activity against MDR *A. baumannii*. These findings support its potential as a biocompatible coating strategy for limiting bacterial colonisation of medical device surfaces. Further, the study was limited to *in vitro* evaluation, which may not fully reflect the physiological and microbial complexity of clinical conditions. The two-phage cocktail may also have limited host-range coverage; incorporating additional phages with complementary host ranges could broaden its activity against diverse *A. baumannii* isolates and other clinically relevant pathogens.

## Conclusion

In this study, a sodium alginate-polyvinyl alcohol (SA-PVA) hydrogel system was used as a coating platform for the localised delivery of a bacteriophage cocktail targeting *A. baumannii*. The formulated hydrogel demonstrated suitable physicochemical characteristics, effective phage encapsulation, and a sustained release profile, facilitating prolonged antibacterial action at the material surface. Notably, the phage- loaded hydrogel coating significantly reduced bacterial biofilm formation, which is a critical factor in the onset and persistence of device-associated infections. The system also demonstrated good cytocompatibility, indicating its suitability for biomedical use. Overall, these findings highlight the promise of SA-PVA hydrogel-based phage delivery systems as a novel and targeted strategy to combat multidrug-resistant pathogens, particularly *A. baumannii*, in medical device-related infections. Further *in vivo* validation studies will be essential for future clinical application.

## Data Availability

The original contributions presented in the study are included in the article/Supplementary material, further inquiries can be directed to the corresponding author.

## Footnotes

1 Bacteriophage cocktail loaded SA-PVA hydrogel.
