# Supplementary material for: Phage cocktail-loaded hydrogel coating prevents the biofilm formation of MDR Acinetobacter baumannii on indwelling medical devices
Source: Front Microbiol. 2026 Oct 30;17:1990693. doi: 10.3389/fmicb.2026.1990693 (PMC13766307; doi:10.3389/fmicb.2026.1990693)
Supplement: Supplementary file 1 [file Supplementary_file_1.docx]

**Supplementary Information:**

**Title of manuscript:** Phage Cocktail-Loaded Hydrogel Coating Prevents the Biofilm Formation of MDR *Acinetobacter baumannii* on Indwelling Medical Devices

**Authors Details:**

Nisha K Rai^1^, Kokkarambath Vannadil Suchithra^1^, Juliet Roshini Mohan Raj^2^, Prakash Patil^3^, Mohana Kumar Basavarajappa^4^, Veena A Shetty^1^

1.Nitte (Deemed to be University), KS Hegde Medical Academy (KSHEMA), Department of Microbiology, Deralakatte, Mangalore -575 018, India

2.Nitte (Deemed to be University), Nitte University Centre for Science Education and Research, Department of Infectious diseases and microbial genomics, Deralakatte, Mangalore -575 018, India

3.Nitte (Deemed to be University), KS Hegde Medical Academy (KSHEMA), Central Research Laboratory, Deralakatte, Mangalore -575 018, India

4.Nitte (Deemed to be University), KS Hegde Medical Academy (KSHEMA), Nitte University Centre for Stem Cell Research and Regenerative Medicine, Deralakatte, Mangalore -575 018, India

| **Characterizations** | **vB_AbaM_561a** | **vB_AbaS_561b** |
| --- | --- | --- |
| Order | Caudovirale | Caudovirale |
| Head size | 82-92nm | 145-149nm |
| Tail size | 49.4nm | 149nm |
| Burst size | 33 PFU/mL | 37 PFU/mL |
| Latent period | 30 min | 40 min |
| Host range | 39% | 36% |
| pH stability | 3-8 pH | 5-8 pH |
| Temperature stability | 4-37˚C | 4-37˚C |

**S1: Basic characterizations of the bacteriophages used in the cocktail formulation**.
